# Total synthesis of insect sex pheromones: recent improvements based on iron-mediated cross-coupling chemistry

**DOI:** 10.3762/bjoc.19.15

**Published:** 2023-02-14

**Authors:** Eric Gayon, Guillaume Lefèvre, Olivier Guerret, Adrien Tintar, Pablo Chourreu

**Affiliations:** 1 M2i Development, Bâtiment ChemStart’Up, 64170 Lacq, France; 2 i-CLeHS, UMR 8060, CNRS Chimie ParisTech 11, rue Pierre et Marie Curie, 75005 Paris, France

**Keywords:** catalysis, cross-coupling, insect pheromones, iron

## Abstract

In the current ecological context, use of insect sex pheromones as an alternative to conventional pesticides is in constant growth. In this report, we discuss the recent contributions brought by our groups in the field of iron-catalyzed cross-couplings applied to the synthesis of insect pheromones. The pivotal question of the development of sustainable synthetic procedures involving cheap, non-toxic and efficient additives is also discussed, as well as the mechanistic features guiding the reactivity of such catalytic systems.

## Introduction

Public health issues related to environmental problems, particularly pollution and soil persistence, led in recent years to a decrease in the use of so-called conventional protection products for the protection of crops and plants. Some of these synthetic pesticides, such dimethoate (pesticides for the protection of cherry crops) or chlordecone (pesticides for the protection of banana crops) [1], have recently been forbidden in the EU. In this context, the development of eco-friendlier solutions for the protection of crops and plants is a major challenge. This is why biocontrol (that is: all plant protection methods that use natural mechanisms to help regulate/balance the populations of harmful species rather than eradicating them) based on the use of insect pheromones [2–5] is an eco-friendly solution for the protection of a wide range of crops and plants against pests. Indeed, those chemical mediators, which are emitted by insects from a given species, trigger a specific reaction, such as sexual attraction, on an individual of the same species and represent very selective intraspecific means of communication. Used in crop protection, synthetic pheromones can disturb communication between individuals in order to prevent their reproduction and then limit their proliferation, finally lowering the damages caused by pests to the crops. Moreover, those molecules are totally biodegradable, harmless to Humans and don’t interact with non-targeted insects [6].

**Scheme 1:**
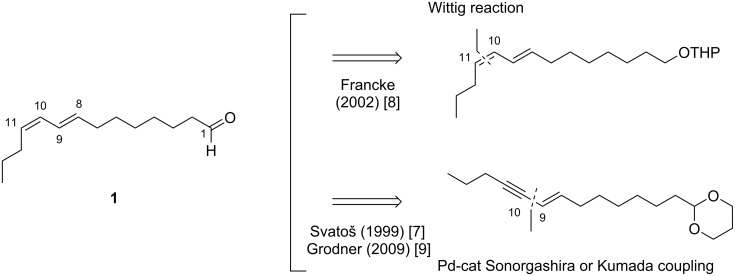
Structure of the (8*E*,10*Z*)-tetradecadienal (**1**, sex pheromone of the horse-chestnut leaf miner) and reported retrosynthetic pathways.

However, at this time, few pathways for the synthesis of insect pheromones reported in the literature are applicable on an industrial scale, which slows the widespread application of this solution. As a matter of fact, the key step of the synthesis of such compounds, which is the introduction of C=C unsaturations, traditionally involves expensive, toxic and low atom economy methodologies. The synthesis of the sex pheromone of the horse-chestnut leaf miner (*Cameraria ohridella*), (8*E*,10*Z*)-tetradecadienal (**1**, Scheme 1) perfectly illustrates this issue: the main synthetic pathways reported in the literature until now (Svatoš, 1999 [7]; Francke, 2002 [8], Grodner, 2009 [9]) are linear syntheses involving a great number of steps and purifications as well as cryogenic temperatures. Moreover, the introduction of the C=C unsaturation is achieved via a Wittig reaction or a Pd-catalyzed Sonogashira cross-coupling followed by a reduction by a borane reagent, methods which lead to overall processes with a low atom economy, generating a significant amount of chemical waste, and which can be expensive when noble metals such as palladium salts are required (Scheme 1).

**Scheme 2:**
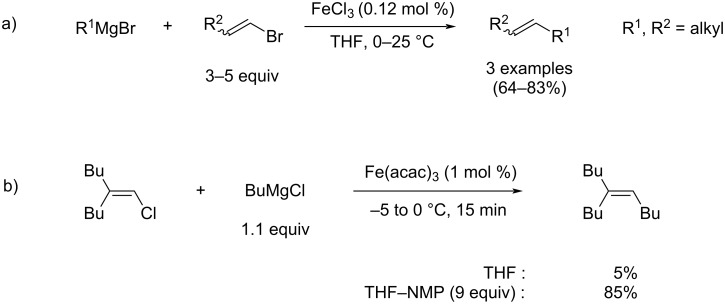
a) Alkyl–vinyl seminal cross-coupling reaction by Kochi; b) improved procedure described by Cahiez.

The high efficiency of transition-metal-catalyzed cross-coupling methods in C–C bond formation processes constitutes an extremely powerful tool in total synthesis. In order to reach sustainable and economically appealing conditions, the quest for the substitution of palladium by non-noble metals has been investigated for more than four decades. To address this issue, new eco-friendly synthetic routes relying on carbon–carbon iron-catalyzed cross coupling as a key step were developed, capitalizing on the low toxicity and the cheap cost of this abundant metal [10]. For instance, in 1971, Kochi developed an iron-catalyzed alkyl–alkenyl cross-coupling reaction between aliphatic Grignard reagents and vinyl bromides, using FeCl_3_ as the catalyst, in order to obtain cross-coupling products with yields between 64 and 83% (Scheme 2a) [11]. Drawing his inspiration from Kochi, Cahiez reported that using *N*-methyl-2-pyrrolidone or NMP as a co-solvent drastically improved the efficiency of the iron-catalyzed alkyl–alkenyl cross coupling reaction developed by Kochi, leading to better yields, with no need to use an excess of one of the coupling partners (Scheme 2b) [12]. The yields obtained using this ligand-free method are comparable to those obtained in recent palladium-mediated alkyl–vinyl cross-couplings using exogenous N- or P-based ligands, highlighting the efficiency of iron as a credible alternative to noble metal catalysis in cross-coupling chemistry [13]. Cahiez’ pioneering work highlighted the potential of iron catalysis in organic chemistry and generated a new interest for the study of iron-catalyzed reactions, which witnessed considerable development in the last two decades [14], several cross-coupling methodologies involving soft nucleophiles, such as iron-mediated Suzuki–Miyaura cross-couplings, being reported [15].

The introduction of alkyl–alkenyl linkage by means of iron-catalyzed cross-coupling reactions thus appears as an appealing tool for the synthesis of insect pheromones, which often display linear chains involving C=C unsaturations with a well-defined stereochemistry (Scheme 1). Therefore, we aimed at developing this strategy for the design of high-scale, eco-friendly and economic new synthetic procedures applied to obtain a variety of insect pheromones. The present report summarizes the recent progresses made in insect sex pheromone synthetic applications in our group, with a particular focus on the quest for non-toxic and sustainable catalytic platforms developed in the iron-catalyzed cross-coupling field.

## Discussion

### The quest for non-toxic and efficient additives for iron-mediated cross-coupling reactions

#### Co-solvents used as NMP surrogates

In spite of the very good yields and soft conditions provided by Cahiez’ method (Scheme 2b), a major drawback of the latter is the requirement of NMP as a co-solvent. Indeed, NMP recently proved to be of very high concern due to its acute toxicity, since it was classified as a reprotoxic reagent [16–17]. In order to circumvent this matter and develop new non-toxic and sustainable iron-mediated coupling transformations, efforts were put in the quest for suitable additives as surrogates of NMP. Szostak and Bisz reported in 2019 that *N*-methyl-ε-caprolactame (NMCPL) could be used as an alternative solvent, and sp^3^–sp^2^ cross-coupling reactions proceeding with good to excellent yields were reported. Alkyl Grignard reagents could thus be used in combination with a variety of alkenyl chlorides and (hetero)aryl chlorides [18].

#### Importance of the leaving groups: use of enol phosphates

In order to overcome the delicate choice of either NMP or one of its surrogates as a suitable co-solvent, it has also been demonstrated that amide-free catalytic procedures could afford satisfying coupling yields by a suitable variation of the nature of the leaving group. In 2008, Cahiez reported an iron-catalyzed alkenylation of organomagnesium reagents with enol phosphates as electrophiles, instead of alkenyl halides [19]. In this case, when reactive enol phosphates derived from aldehydes were used, excellent coupling yields were obtained in THF, with no need of NMP additive (Table 1, entries 1 and 2). However, less reactive α,β-disubstituted enol phosphates required the presence of NMP (Table 1, entry 3) or even *N*,*N*′-dimethylpropyleneurea (DMPU) (Table 1, entry 4) as a co-solvent.

**Table 1:** Iron-catalyzed alkenylation of enol phosphates by Grignard reagents by Cahiez.

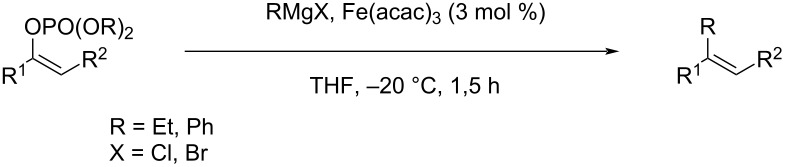				

Entry	Enol phosphate (*E*:*Z*)	Product (*E*:*Z*)	Solvent	Yield (%)

1	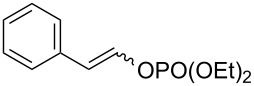 (86:14)	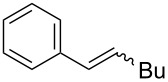 (90:10)	THF	87
2	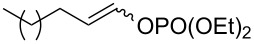 (70:30)	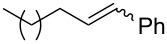 (85:15)	THF	81
3	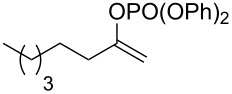	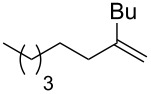	THF + NMP	75
4	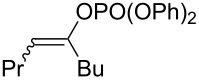 (68:32)	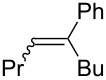 (94:6)	THF + DMPU	70

**Scheme 3:**
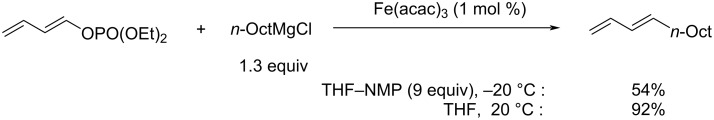
Iron-catalyzed cross-coupling of *n-*OctMgCl with a 1-butadienyl phosphate.

Nevertheless, capitalizing on these results, Cahiez further developed this iron-catalyzed cross coupling reaction with dienol phosphates as electrophiles, which are thermally more stable than dienyl halides [20]. It was found that when dienol phosphates were used as electrophiles, the presence of NMP is actually detrimental. Indeed, for the cross coupling reaction of octylmagnesium chloride with 1-butadienyl phosphate in THF at −20°C in the presence of 1% Fe(acac)_3_ and 9 equivalents of NMP, the resulting 1,3-dodecadiene cross-coupling product was only obtained with a 54% yield, whereas when the reaction was performed in THF alone (at 20 °C), the yield improved significantly (92%, Scheme 3).

**Scheme 4:**
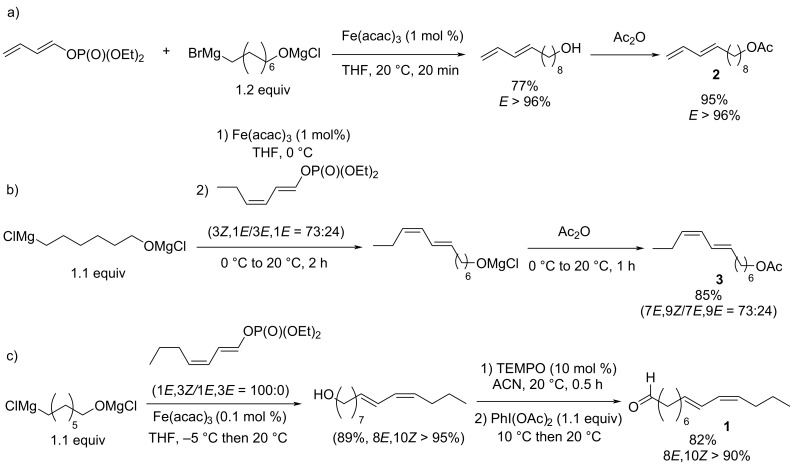
Synthesis of several insect sex pheromones (a) red bollworm moth, b) European grapevine moth, c) horse-chestnut leaf miner) involving a key alkyl–alkenyl iron-mediated cross-coupling between a dienol phosphate and an α,ω-difunctionalized Grignard reagent.

The efficient iron-mediated alkylation of dienyl phosphates, proceeding with an excellent retention of stereochemistry, was then successfully used in the introduction of the alkyl–alkenyl linkage of several insect pheromones. Cahiez paved the way of this strategy in 2008, showing that (*E*)-dodeca-9,11-dien-1-yl acetate (**2**), the sex pheromone of red bollworm moth (*Diparopsis castanea*), which contains a terminal diene, could be obtained at a laboratory scale (ca. 200 mg) by means of iron-mediated cross-coupling (77% yield for the coupling step, Scheme 4a) [20]. In this cross-coupling, an α,ω-difunctionalized Grignard reagent bearing a magnesium alkoxide moiety at the end of the aliphatic chain is used as a coupling partner. Following in those footsteps, similar syntheses of other insect pheromones involving a key alkyl–alkenyl linkage introduction by iron-catalyzed cross-coupling reactions of α,ω-difunctionalized alkyl Grignard reagents with stereochemically pure dienyl phosphates were successfully performed at higher scales, in order to be successfully implemented in industrial processes. (7*E*,9*Z*)-Dodeca-7,9-dien-1-yl acetate (**3**), the sex pheromone of European grapevine moth (*Lobesia botrana*), was thus obtained at a 14 g scale, with an overall 85% yield for the one-pot sequence involving the iron-catalyzed cross coupling followed by acetylation with Ac_2_O (Scheme 4b) [21]. Illustrating the applicability of this process to large industrial scales, a synthesis of 50 kg of pheromone **3** was performed using this method [22–23]. Similarly, a 40 g batch of (8*E*,10*Z*)-tetradeca-8,10-dienal (**1**), the sex pheromone of horse-chestnut leaf miner (*Cameraria ohridella*) was obtained following an analogous procedure (Scheme 4c) [24]. It is of note that a low 0.1 mol % catalytic loading could be used for the latter coupling, which is thus particularly appealing for high-scale applications. In this context, the procedure described in Scheme 4c is currently used for industrial medium-scale synthesis of **1** by the M2i Company.

#### Magnesium alkoxides used as additives

Although it can lead to efficient NMP-free cross-coupling procedures, the use of enol phosphates as coupling electrophiles also brings several issues for efficient and sustainable procedures meant to be carried out at large scales. First, dialkyl phosphates used as leaving groups lead to overall transformations which display a poor atom economy. Moreover, diethyl chlorophosphate (DCP), used as a classic starting material in the dienol phosphates synthesis, is also registered under REACH regulation, and its use brings several toxicity issues for living organisms [25–26]. Additionally, the classic stereoselective synthesis of dienol phosphates requires cryogenic temperatures (−78 °C) as well as NMP-based methodologies [27]. Therefore, motivated by those considerations, we sought a suitable efficient and non-toxic additive which would allow the use of alkenyl halides as cross-coupling partners in the absence of NMP.

A remarkable feature of the alkyl–dienyl cross-coupling reactions displayed in Scheme 4 is the implication of α,ω-difunctionalized Grignard reagents, bearing a magnesium alkoxide moiety at the end of the chain. Magnesium alkoxides exhibit strong σ-donating properties, given the high nucleophilicity of the alkoxide moiety. Moreover, the beneficial effect of molecular σ-donating additives on the yields and selectivities of iron-mediated cross-coupling reactions had already been reported in the literature. Nakamura thus described an elegant aryl–aryl cross-coupling procedure suppressing the formation of Grignard homocoupling byproducts relying on the use of FeF_3_ as catalyst, associated with strong N-heterocyclic carbenes (NHCs) and a source of fluoride anions [28]. A similar procedure involving sodium alkoxide additives and NHCs was also described by Duong for the aryl–aryl cross coupling [29]. Alkoxide salts appear as good alternatives to NMP or phosphate-based additives, since several classic alcohol sources display low toxicities and can also come from renewable resources [30].

**Scheme 5:**
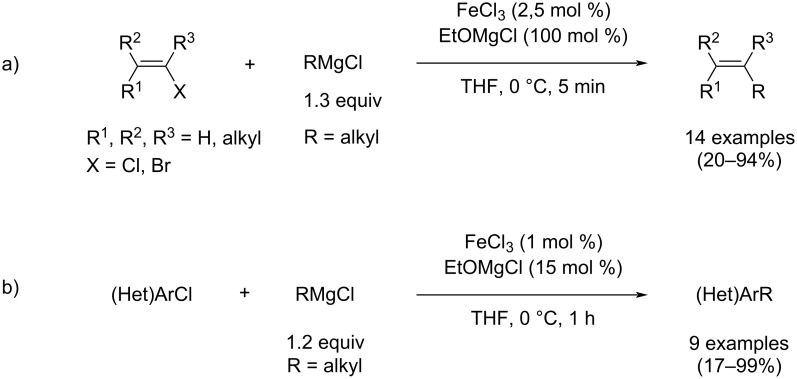
Cross-coupling of alkyl Grignard reagents with a) alkenyl or b) aryl halides involving EtOMgCl as additive.

In this context, Cahiez and Lefèvre developed an eco-friendlier, and easily scalable iron-catalyzed cross-coupling method between alkyl Grignard reagents and C_sp2_ (alkenyl or aryl) organic halides [31]. By combining a catalytic charge of FeCl_3_ with alkoxide magnesium salts such as EtOMgCl in THF, complete conversion of the alkenyl/aryl halides was observed and the cross-coupling products were afforded in good to excellent yields (Scheme 5). Moreover, this reaction can also be carried out at the gram scale (up to 50 mmol for alkyl–alkenyl coupling reactions, up to 10 mmol for alkyl–aryl couplings).

Alkoxide salts, such as EtOMgCl, are cheaper and much less toxic than NMP, making this method particularly eco-friendly. Moreover, this method also provides results equivalent to those obtained using NMP-based cross-coupling reactions. Again, no exogenous nor expensive ligands are required, leading to an overall economically viable process. Those results clearly demonstrate that magnesium alkoxide additives per se can play a beneficial role in alkyl–alkenyl iron-mediated cross-coupling reactions, similarly to the results observed when NMP is used as a co-solvent (Scheme 2b).

Therefore, targeting applications in pheromone synthesis, we aimed at combining the advantages brought by those additives with the atom-economy offered by the use of organic electrophiles bearing halide leaving groups (better than that obtained with phosphates leaving groups, Scheme 4). We thus investigated the development of pheromone synthesis involving cross-coupling methods using Grignard reagents bearing terminal alkoxide magnesium moieties and dienyl halides. This strategy is moreover particularly appealing since the magnesium alkoxide additive used in the cross-coupling is also a part of the final synthetic target. The oxygen atom of the -OMgX functionality indeed affords the terminal oxidized function of the pheromone molecule (alkoxy acetate in **2** or **3**, Scheme 4a and 4b, or formyl group in **1**, Scheme 4c).

**Scheme 6:**
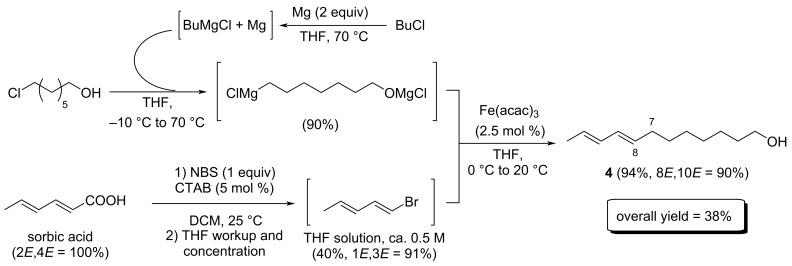
Total synthesis of codling moth sex pheromone **4** using an iron-mediated cross-coupling between an α,ω-difunctionalized Grignard reagent and a dienyl bromide.

As a representative target, we developed the total synthesis of the codling moth sex pheromone, (8*E*,10*E*)-dodecadien-1-ol (**4**), featuring the introduction of the C_7_–C_8_ linkage by a key iron-mediated cross-coupling sequence between the suitable α,ω-difunctionalized Grignard reagent and 1-bromopenta-1,3-diene as the electrophile (Scheme 6) [32].

A classic drawback of the use of dienyl halides as coupling partners is their intrinsic thermal instability. Dienyl halides indeed tend to polymerize at low temperatures, and drastic storage conditions must be applied. For example, chloroprene, a well-known precursor of isoprene polymers, must be transported under inert atmosphere below −10 °C, and an inhibitor must be added to prevent polymerization. Cross-coupling systems involving this reagent are thus particularly scarce, and few examples are reported, using for some of them Ni-based catalysts [33].

1-Bromopenta-1,3-diene used in the synthesis of **4** (Scheme 6) is not an exception. The expected (*E*,*E*) isomer was easily obtained from sorbic acid, a renewable C_6_ synthon, by a Hunsdiecker–Borodin bromodecarboxylation, adapting the recent micellar conditions developed by Rajanna for the synthesis of alkenyl halides starting from α,β-unsaturated acids [34]. However, purification and concentration of 1-bromopenta-1,3-diene proved to only promote its degradation, likely by polymerization. To circumvent this matter, we developed a workup procedure allowing to directly obtain THF solutions of 1-bromopenta-1,3-diene in the 0.4–0.7 M concentration range, and no further purification was required. Those solutions can be directly used in a subsequent iron-mediated cross-coupling procedure. We demonstrated that the alkyl–dienyl cross coupling of those solutions of 1-bromopenta-1,3-diene with α,ω-difunctionalized Grignard reagents afforded the coupling product **4** with an excellent 94% yield and a total retention of stereochemistry (Scheme 6). Overall, sex pheromone **4** can be obtained in a convergent total synthesis with a key iron-mediated cross coupling starting from sorbic acid and a commercially available α,ω-chloroalcohol in 4 steps, with an overall 38% yield.

This method can also be used for the alkylation of dienyl bromides by classic aliphatic or aromatic Grignard reagents which do not bear ω-magnesium alkoxide functionalities: in that case, EtOMgX can be used as an external additive to enhance the cross-coupling selectivity, in line with the alkyl–alkenyl and aryl–alkenyl cross-coupling methods discussed earlier (Scheme 5) [32].

The beneficial role of magnesium alkoxide salts in the outcome of iron-mediated alkyl–alkenyl cross-coupling reactions can thus display a double interest for the total synthesis of insect pheromones. Indeed, α,ω-difunctionalized Grignard reagents featuring a terminal magnesium alkoxide group can be used as both nucleophilic partners and as a magnesium alkoxide additive source, enabling the development of iron-mediated cross-coupling procedures with alkenyl or dienyl halides, requiring neither heavy alkyl phosphate leaving groups nor toxic solvents such as NMP. Moreover, those procedures can be carried out at large industrial kilogram scales, leading to green, efficient and economically viable processes.

### Mechanistic considerations

The improvements of the initial cross-coupling conditions developed by Kochi (Scheme 2a) discussed in this report have high potent synthetic applications since they do not require well-defined iron precursors stabilized by highly functionalized ligands, the high price of the latter often precluding their use in high-scale syntheses. On the other hand, the exact role played at a molecular level by the additives discussed herein (NMP or one of its surrogates, magnesium alkoxides, phosphate leaving groups, etc.) is still an unclear question, even if the latter species proved to have an unambiguous beneficial effect on the cross-coupling yields and selectivities. Several recent literature results which enlighten the role of those additives, either brought by external reagents or by in situ-formed intermediates, are gathered in Table 2.

**Table 2:** Selected additives used in iron-mediated cross-coupling procedures and their role at a molecular level.

	Nucleophile	Electrophile	Additive	Molecular effect of the additive	Reference

1	MeMgBr	PhCH=CHBr	NMP	formation of [Mg(NMP)_6_]^2+^; stabilization of [Me_3_Fe^II^]^−^	[12,35]
2	AlkylMgBr ArMgBr	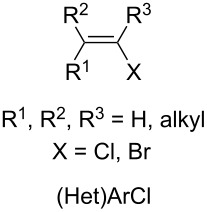	EtOMgX	formation of [Mg]-OEt adducts, stabilization of [Me_3_Fe^II^]^−^ (high RMgX:EtOMgX ratio); and/or [Fe^II^]-OEt intermediates (low RMgX:EtOMgX ratio)	[38]
3	AlkylMnBr MesMgBr	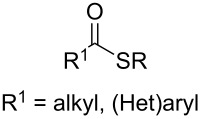	RS^–^	formation of Mes-[Fe^II^]-SEt adducts	[39]
4	AlkylMgBr	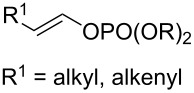	phosphate group	unknown	[20–24]

The beneficial role of the NMP co-solvent on the alkyl–alkenyl cross-coupling yield reported by Cahiez (Scheme 2b) and considerably used ever since was explained at a molecular level by Neidig in 2018. It was demonstrated that NMP did not act as a ligand to the iron center in alkyl–alkenyl cross-coupling reactions featuring Grignard reagents, but was involved in the formation of the bulky dication [Mg(NMP)_6_]^2+^, the presence of the latter contributing to the kinetic stabilization of the on-cycle active species [Me_3_Fe^II^]^−^ (Table 2, entry 1) [35]. The exact nature of the interaction between the active *ate*-ferrous complex and the NMP-ligated magnesium cation is so far unknown. Although often neglected in the mechanistic analysis of a cross-coupling system, the importance of the coordination sphere of the main-group cation brought by the nucleophilic partner R[M] in a cross-coupling has been reported in the recent literature. It was indeed shown that some ligands (such as diphosphines) used in Fe-catalyzed Negishi cross-coupling reactions (R[M] = RZnX) were actually involved in the coordination of the Zn^II^ cation in a key on-cycle transmetallation step, and not in the coordination of the iron-containing intermediates [36–37].

The use of magnesium alkoxide salts, either as an ω-functionalization of the nucleophilic partner (Scheme 4) or as an external molecular additive such as EtOMgCl (Scheme 5), also likely proceeds similarly to the NMP, as demonstrated by recent studies by Neidig and Lefèvre (Table 2, entry 2) [38]. No alteration of the structure of the active species [Me_3_Fe^II^]^−^ is observed in the presence of an excess of EtOMgCl, suggesting that the alkoxide additives solely bind to the magnesium cations. However, for very low RMgX:EtOMgX ratios, minor species possibly featuring alkoxide stabilized ferrous [Fe^II^]-OEt intermediates were observed by paramagnetic ^1^H NMR spectroscopy.

In line with this result, Fleischer and Lefèvre also recently demonstrated that the anionic thiolate leaving group EtS^–^ released in Fukuyama cross-coupling reactions between alkylmanganese nucleophiles AlkylMnX and thioesters RC(O)SEt using Fe(acac)_3_ as catalyst could act as a ligand to on-cycle organoiron(II) intermediates (Table 2, entry 3) [39]. This behaviour opens the door to interesting synthetic perspectives, since it suggests that leaving groups can act as stabilizing additives to on-cycle organometallic intermediates in cross-coupling procedures. Given that the quantity of free anionic leaving groups, released at each turnover cycle, increases upon completion of the coupling process, it means that the catalyst resting state strongly benefits from the leaving group ligation at the latest stages of the cross coupling. This is particularly interesting since it can hamper the usual decomposition of the iron catalyst, which tends to afford unreactive reduced aggregates at the end of the catalytic transformation, when the coupling kinetics is slowed down due to the drop of the concentration of coupling reagents. The affinity of ferrous precatalysts for thiolate ligands had also been reported by Cahiez earlier in the cross coupling of alkyl Grignard reagents with alkenyl halides, 2-naphthylthiolates being used as additives [40]. No in-depth mechanistic studies were so far reported regarding the reactivity of the enol phosphate electrophiles (Scheme 3 and Scheme 4, and Table 2, entry 4). Phosphate free anions released at each catalytic cycle could act either as NMP or alkoxides, that is, as ligands to the magnesium cations, or as thiolate leaving groups, and stabilize at a molecular level active Fe^II^ species. In both cases, a beneficial effect should be observed at a molecular level on the stabilization and/or the reactivity of on-cycle species, given that phosphate electrophiles proved to afford satisfying coupling yields in the absence of other additives.

## Conclusion

In conclusion, iron-mediated cross-coupling reactions between Grignard reagents and alkenyl organic electrophiles prove to be an efficient method for the introduction of the key alkyl–alkenyl linkages in insect sex pheromones total synthesis. This method has been applied to a variety of targets, and has witnessed important improvements since the seminal reports by Cahiez and Kochi. To date, alternatives to the use of reprotoxic additives such as NMP have been reported (involving, e.g., phosphate leaving groups, or magnesium alkoxide additives), and can be efficiently applied at large industrial scales.

Extension of such methods to more challenging hybridization patterns in cross-coupling chemistry applied to synthesis of other insect pheromones is currently investigated in our groups.
